# Predicting Clinical Outcomes at the Toronto General Hospital Transitional Pain Service via the Manage My Pain App: Machine Learning Approach

**DOI:** 10.2196/67178

**Published:** 2025-03-28

**Authors:** James Skoric, Anna M Lomanowska, Tahir Janmohamed, Heather Lumsden-Ruegg, Joel Katz, Hance Clarke, Quazi Abidur Rahman

**Affiliations:** 1 Department of Electrical and Computer Engineering, McGill University, Montreal, QC, Canada; 2 ManagingLife, Toronto, ON, Canada; 3 Transitional Pain Service, Department of Anesthesia and Pain Management, Toronto General Hospital, University Health Network, Toronto, ON, Canada; 4 Department of Psychology, York University, Toronto, ON, Canada; 5 Department of Anesthesiology and Pain Medicine, University of Toronto, Toronto, ON, Canada; 6 Department of Computer Science, Trent University, 1600 West Bank Drive, Peterborough, ON, K9L 0G2, Canada, 1 (705) 748-1011 ext 7854

**Keywords:** chronic pain, transitional pain, pain interference, machine learning, prediction model, manage my pain, pain app, clinical outcome, Toronto, Canada, transitional pain service, pain service, pain, app, application, prognosis, chronic pain management, digital health, digital health tool, pain management, machine learning methods, machine learning, prediction, machine learning models, logistic regression

## Abstract

**Background:**

Chronic pain is a complex condition that affects more than a quarter of people worldwide. The development and progression of chronic pain are unique to each individual due to the contribution of interacting biological, psychological, and social factors. The subjective nature of the experience of chronic pain can make its clinical assessment and prognosis challenging. Personalized digital health apps, such as Manage My Pain (MMP), are popular pain self-tracking tools that can also be leveraged by clinicians to support patients. Recent advances in machine learning technologies open an opportunity to use data collected in pain apps to make predictions about a patient’s prognosis.

**Objective:**

This study applies machine learning methods using real-world user data from the MMP app to predict clinically significant improvements in pain-related outcomes among patients at the Toronto General Hospital Transitional Pain Service.

**Methods:**

Information entered into the MMP app by 160 Transitional Pain Service patients over a 1-month period, including profile information, pain records, daily reflections, and clinical questionnaire responses, was used to extract 245 relevant variables, referred to as features, for use in a machine learning model. The machine learning model was developed using logistic regression with recursive feature elimination to predict clinically significant improvements in pain-related pain interference, assessed by the PROMIS Pain Interference 8a v1.0 questionnaire. The model was tuned and the important features were selected using the 10-fold cross-validation method. Leave-one-out cross-validation was used to test the model’s performance.

**Results:**

The model predicted patient improvement in pain interference with 79% accuracy and an area under the receiver operating characteristic curve of 0.82. It showed balanced class accuracies between improved and nonimproved patients, with a sensitivity of 0.76 and a specificity of 0.82. Feature importance analysis indicated that all MMP app data, not just clinical questionnaire responses, were key to classifying patient improvement.

**Conclusions:**

This study demonstrates that data from a digital health app can be integrated with clinical questionnaire responses in a machine learning model to effectively predict which chronic pain patients will show clinically significant improvement. The findings emphasize the potential of machine learning methods in real-world clinical settings to improve personalized treatment plans and patient outcomes.

## Introduction

Chronic pain affects more than a quarter of people worldwide and carries substantial personal and economic burdens [1]. It is a complex condition that can be challenging to assess clinically due to the individualized and subjective nature of symptoms [2,3]. The development and progression of chronic pain are unique to each patient and difficult to predict at the individual level due to the contribution of interacting biological, psychological, and social factors [1,4-7]. Physiological markers associated with chronic pain can provide useful insights about a patient’s condition [8]; however, clinical assessment of the subjective magnitude of pain severity and its impact on function and quality of life primarily relies on self-report measures [4,9]. Self-tracking of symptoms, medications, and daily activities is a popular approach to providing insights about symptom trends in chronic conditions [10,11], and digital apps have become a particularly useful tool to support self-tracking of pain and related symptoms [12-14]. With recent advances in machine learning technologies, digital symptom tracking opens an opportunity to evaluate numerous factors that contribute to pain symptoms and functioning to make predictions about an individual patient’s progress [12]. This work examines whether information obtained from a pain tracking app can be used to accurately predict improvement in clinical pain-related outcomes in patients at a transitional pain clinic.

Manage My Pain (MMP) is a digital health app designed by ManagingLife with a patient-centric approach, aimed at helping patients and health care professionals measure, manage, and communicate pain, function, and medication use both at home and in clinical settings. The app has over 100,000 users worldwide and is available in 7 languages on both mobile and web platforms. MMP has been integrated into the Transitional Pain Service (TPS), a multidisciplinary pain clinic at Toronto General Hospital (TGH), to support symptom assessment and patient engagement with symptom tracking [13]. The TPS at TGH is a pioneering clinic that treats patients during the transitional period when acute pain after a major surgical procedure has the risk of becoming chronic [15,16]. The clinic also treats complex patients with chronic pain to support them in medication management and opioid weaning [17]. With this patient population, the clinic relies on regular symptom tracking to monitor patient progress and to intervene appropriately during critical phases of pain treatment. The MMP app provides a comprehensive digital platform where patients can fill out intake and follow-up questionnaires, track their symptoms, view symptom patterns and trends, and access educational resources about managing pain. Clinicians, in turn, can view each patient’s record in the app to gain insights into their ongoing pain and symptom patterns and to support patient-clinician communication and decision-making about treatment. The MMP app has become the standard of care at the TPS since May 2020. Evaluations of the app showed that it was acceptable to TPS patients [18] and patients who used it reported significantly lower anxiety and pain catastrophizing scores [19].

The aim of this study is to use machine learning techniques to predict clinically significant improvements in pain-related outcomes among TPS patients who used the MMP app. In previous studies, we used symptom tracking, profile, and usage data from users of the MMP app to predict variability in reported pain levels over time (ie, pain volatility) [20,21]. In this study, we focused on a population of users from the TPS clinic and incorporated data from clinical questionnaires alongside symptom tracking, profile, and use data to predict clinical outcomes related to pain interference. Pain interference refers to the impact pain has on engagement in daily activities and participation [22]. It is related to the perceived severity of pain [23] and is considered a key aspect of the pain experience and a primary outcome in many clinical trials [24]. It is an informative measure from a clinical treatment and pain management standpoint that focuses on patients’ daily functioning rather than pain intensity itself [25-27]. It is therefore a valuable measure to consider in predicting meaningful improvement for patient outcomes. We hypothesize that a machine learning model using data entered by TPS patients into the MMP app during the first 30 days of use can accurately predict subsequent pain interference scores reported within the next 5-month period.

## Methods

### Ethical Considerations

The study protocol was reviewed and approved by the McGill University Research Ethics Board (File Number 23-12-016). Informed consent to the use of data was obtained when users registered an account through the MMP app and agreed to its End User Licence Agreement [28]. Privacy and confidentiality of user data were protected in accordance with ManagingLife’s Privacy Policy [29]. All user data used in the study dataset was deidentified. Users did not receive compensation for the use of their data in the study.

### Manage My Pain App

**Figure 1.**
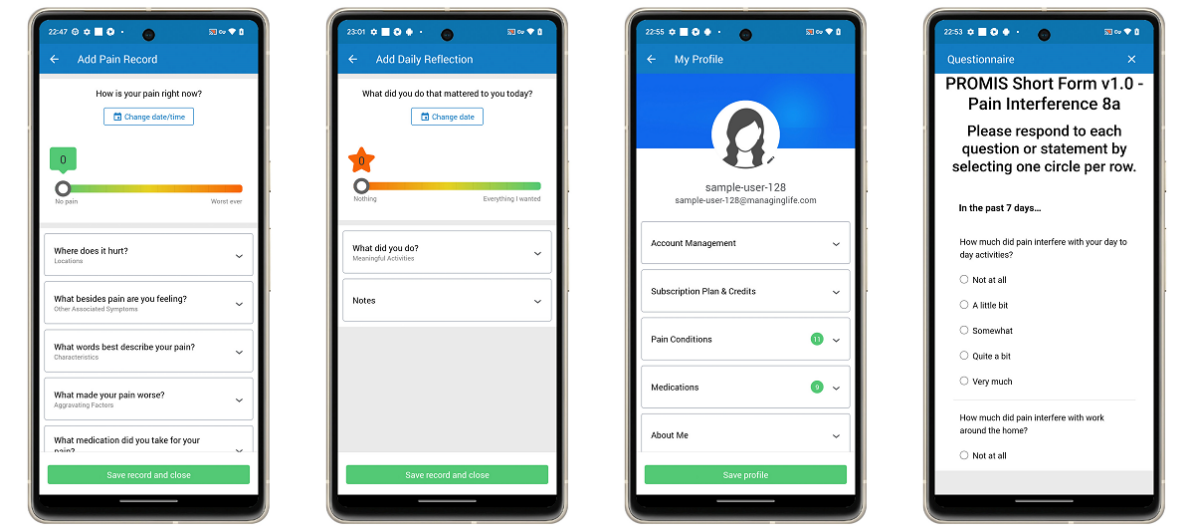
Screenshots of the mobile version of the Manage My Pain (MMP) app. Shown from left to right is the interface of the pain record, daily reflection, user profile, and clinic questionnaire.

The MMP app is available on Android, iOS, and web devices. The main features of the app are the daily reflection and the pain record that allows in-the-moment logging of pain experiences. Initially, users interact with the MMP app by responding to a daily push notification that prompts them to reflect on their day and rate it by completing a daily reflection at a default time of 8 PM. Users have the option to customize or disable the timing and frequency of notifications. The daily reflection, based on Acceptance and Commitment Therapy principles known for their efficacy in chronic pain management [30,31], asks users, “What did you do that mattered to you today?” Users then rate their day on a visual analog scale from 0 (Nothing) to 10 (Everything I wanted) and can record any meaningful activities. Following the daily reflection, users are invited to complete a pain record where they assess their current pain level by responding to “How is your pain right now?” and rating it on a scale from 0 (No pain) to 10 (Worst ever). Additionally, users can detail up to 7 aspects of their pain episodes, including body location, symptoms, characteristics, aggravating factors, medications, interventions, and environment. There is no limit to the number of pain records a user can enter in a day and these entries are independent of completing a daily reflection. Users can also enhance their MMP app profile with personal information about their medications, health conditions, and demographics such as age, height, weight, and gender. The screen interface of each of the different features of the MMP app is shown in Figure 1.

### TPS Clinic Patient Flow

The TPS treats patients who are at risk of developing chronic pain after surgery and patients with chronic pain who have complex needs. Patients are typically referred to the TPS during the perioperative period, before or after a surgical procedure, or they are referred through the Toronto Academic Pain Medicine Institute [32]. Prior to their initial assessment by a TPS physician, patients are asked to fill out a set of clinical intake questionnaires (see below for details). Patients are invited to access the questionnaires on the MMP app and staff at the clinic support patients in accessing the app and registering an MMP account. They are also informed about the other features available in the app to track their pain and daily activities, view symptom patterns and trends, and learn about managing pain in the Pain Guide. The TPS clinical team works with patients to address their needs through a multidisciplinary approach to pain care that includes medical treatment alongside psychological care and physical rehabilitation. Patients are asked to fill out follow-up clinical questionnaires using the MMP app at subsequent visits to the clinic. Patients are followed by the TPS for up to 6 months, at which point they are typically discharged to primary care.

### Clinic Questionnaires

The TPS clinic uses a battery of standard clinical questionnaires to evaluate patient pain-related symptoms at intake and to assess treatment progress over time. The following questionnaires are regularly assigned using the MMP app at both intake and follow-up visits and are common across all TPS patients: Numeric Rating Scale (NRS) to rate pain severity on an 11-point scale [9]; PROMIS Pain Interference 8a v1.0 (PROMIS PI) to measure the extent to which pain hinders an individual’s engagement with physical, mental, cognitive, emotional, recreational, and social activities [22]; Pain Catastrophizing Scale (PCS) to assess catastrophic thinking related to pain [33]; Generalized Anxiety Disorder-7 (GAD-7) as a measure of general anxiety [34]; and Patient Health Questionnaire-9 (PHQ-9) as a measure for screening, diagnosing, monitoring, and measuring the severity of depression [35]. Additional questionnaires are assigned as needed to meet the specific needs of each patient.

### Study Dataset

**Figure 2.**
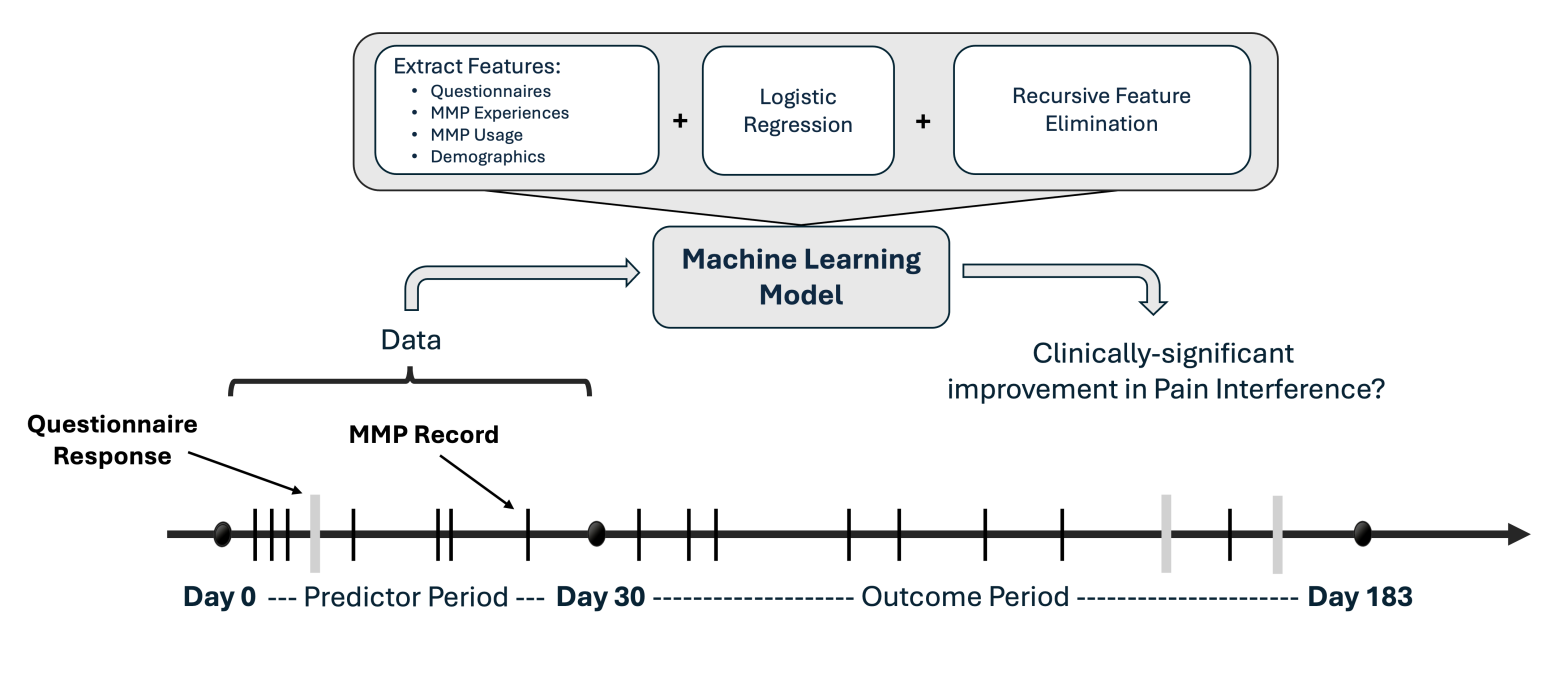
Overview of study timelines and machine learning model approach. MMP: Manage My Pain.

A total of 780 TGH TPS patients entered responses to clinic questionnaires and pain experience records in the MMP app during the period between May, 2020 and March, 2024, producing 14,127 questionnaire responses and 30,033 pain experience records. For this study, we selected users who had at least 1 PROMIS PI questionnaire response in a predictor period, and at least 1 PROMIS PI questionnaire response in an outcome period, resulting in 160 users. The predictor period was defined as the first 30 days of MMP app use, and the outcome period was set between 30 days and 6 months (183 d) from the first app use (Figure 2). There were 680 users who recorded a response in the predictor period, 182 users who recorded a response in the outcome period, and 180 users who had a response in both the predictor and outcome periods. Therefore, 180 users were selected for this study.

We aimed to predict users who improved based on changes in their final score on the PROMIS PI questionnaire between the predictor and outcome period. The final score on the PROMIS PI questionnaire is generated by converting the total raw score into a T-score for each participant using a web-based calculation tool [22]. The first questionnaire responses from the predictor period and the first subsequent recorded responses in the outcome period were used in the prediction model.

Clinical questionnaires have a research-backed minimal clinically important difference (MCID) indicator that guides clinicians in evaluating the progress of symptoms [36]. The MCID was used in this study as a marker of patient improvement. The MCID for the PROMIS PI questionnaire is 2 [37]. Patients who showed an improvement in PROMIS PI questionnaire T-scores greater than the MCID of 2 were classified as improved.

### Data Preprocessing and Feature Extraction

#### Overview

The data were preprocessed to remove any anomalous MMP app records (eg, missing values where entries were expected) and to convert any categorical questionnaire responses into a numerical format.

A total of 245 relevant variables, referred to as features in the field of machine learning, were extracted from the available data. A total of 194 features were extracted from the patients’ MMP app user profiles, pain records, daily reflections, and app use records. Another 51 features were extracted from the patients’ questionnaire responses. Instances with missing values were imputed using the mean value for each feature and subsequently, each feature was *z* score normalized. The features were divided into 8 categories as provided below.

#### Demographics

Demographics (6 features) consisted of data on gender, age, and age category (unknown, 0 to 19, 20 to 29, 30 to 39, 40 to 49, 50 to 59, 60 to 69, and 70+ years), height, weight, and body mass index entered by users into their profiles.

#### Medications

Medications (10 features) consisted of the total number of medications reported by users in their profile and 9 binary features for the specific medications reported, including opioids, tricyclic antidepressants, anticonvulsants, cannabinoids, serotonin-norepinephrine reuptake inhibitors, nonsteroidal anti-inflammatory drugs, acetaminophen, metamizole, and benzodiazepines.

#### Health Conditions

Health Conditions (9 features) consisted of the number of health conditions reported by users in their profile, and the number of conditions by category (unknown, 1 condition, 2 conditions, 3 conditions, and more than 3 conditions). Five features included binary categories of whether a user reported or not one of the following most observed health conditions: fibromyalgia, headaches or migraines, back pain, arthritis, or depression/anxiety. One feature represented an indication of neuropathic pain determined by the app and characterized as the presence of reports of sensations of pins and needles or tingling, burning, numbness, electric shocks, or an aggravating factor of light touch or clothing. The final feature was based on the presence of mental health issues as indicated by reports of anxiety, depression, negative mood, or stress.

#### Pain Record Statistics

Pain Record Statistics (11 features) consisted of the mean and SD of pain severity ratings, the mean and SD of absolute values of changes between consecutive severity ratings, the average of pain ratings in the following categories: mild (average pain rating <4), moderate (average pain rating ≥4 to ≤7), or severe (average pain rating >7), the mean and SD of pain, the number of pain records in the predictor period, the slope of the trendline of the severity scores, and the absolute value of the slope of the trendline.

#### Pain Descriptors

Pain Descriptors (127 features) consisted of descriptors of the pain experience entered into the app, including body locations (32 features), symptoms (21 features), characteristics (21 features), environment (8 features), aggravating factors (15 features), effective factors (15 features), and ineffective factors (15 features).

#### Daily Reflections

Daily Reflections (21 features) consisted of the mean and SD of the daily reflection score, the number of daily reflections in the predictor period, and descriptors of meaningful activities that contributed to the daily reflection rating.

#### App Usage

App Usage (10 features) consisted of the number of completed sections in the user profile, the number of days with a pain record, the percent of descriptor elements that were completed in their pain records, whether users were referred to the app via an institution, provider, or payer, and the average hour, day, week, or month of their pain records. Please note that in the current dataset, all users were referred to the app via an institution.

#### Questionnaires

Questionnaires (51 features) consisted of responses and outcome scores on five clinic questionnaires regularly assigned to all TPS clinic patients at both intake and follow-up visits, including (1) PROMIS PI, 8 questions and 3 outcome scores; (2) NRS, 4 questions and 4 outcome scores; (3) PHQ-9, 9 questions and one outcome score; (4) PCS, 13 questions and one outcome score; and (5) GAD-7, 7 questions and one outcome score. Please refer to Multimedia Appendix 1 to see the questions included in each questionnaire.

### Prediction Model

Given the relatively small sample size, we used binary logistic regression as it is a simple and straightforward model that has been shown to have good performance with limited datasets [38]. Binary logistic regression is a method used for binary classification, which models the probability of each class as a function of the input variables [38]. It operates by fitting a logistic function to the data. The output of logistic regression lies between 0 and 1, representing the probability that a given input point belongs to the class labeled as 1 (improved class). This is achieved by calculating a linear combination of the input features passed through the logistic function. The coefficients are learned by minimizing a cost function. To keep the weights constrained to a reasonable size and to reduce overfitting, we added an L2 regularization term to the cost function which penalizes the scale of the class weights. Additionally, we balanced the weighting of each class inversely proportional to the class frequencies to correct for class imbalance. The model was implemented with the sklearn library [39] in Python, with the liblinear solver [40], as it performs well on smaller datasets.

We then performed feature selection to identify the significant features and improve the model’s generalizability by reducing overfit to the training data. We implemented a recursive feature elimination (RFE) with cross-validation [41]. RFE is a method to identify important features influencing a model’s prediction by systematically eliminating the least important features. First, the training dataset is split into 10 train/test subsets using 10-fold cross-validation, ensuring that the feature elimination process is validated across different subsets of data for reliability. Starting with all features, a logistic regression model is trained, and its performance is evaluated on the validation set. Features are ranked based on their importance from their coefficients, and the least important feature is removed from the set. This process is repeated iteratively, each time removing the least important feature, and the model’s performance is assessed with cross-validation at each step. The optimal number of features is determined by the point where the model’s cross-validation performance is the highest. We used the area under the receiver operating characteristic curve (AUC) as a metric to determine performance. We then implemented a second 10-fold cross-validation to optimize the regularization strength in the logistic regression model using only the selected features.

### Model Evaluation

The model algorithm was validated using leave-one-out cross-validation to assess how well the model will perform in practice on unseen data. In this approach, data from one MMP user is used as the test set while data from the remainder of the users is used as the training set. This process is iteratively repeated such that each subject is used exactly once as the test instance. In each iteration, the entire proposed algorithm is repeated with only the data in the training set being used to train the model. This method allows the model to be evaluated on every possible training and test set combination, providing a comprehensive measure of how well the model performs across the entire dataset. We evaluated the model using 4 metrics as follows: the overall accuracy, the accuracy of the improved class (sensitivity), the accuracy of the not-improved class (specificity), and the AUC. Due to the novel nature of both the dataset and the approach, there are currently no standards to compare against. Therefore, we also evaluated using 3 other standard machine learning models in place of the logistic regression model to compare performance: AdaBoost, random forest, and linear support vector machine (SVM). AdaBoost is an ensemble learning algorithm that iteratively combines weak classifiers to improve overall accuracy by focusing on misclassified instances [42]. Random forest, another ensemble method, builds multiple decision trees and combines their predictions, offering robustness to overfitting and the ability to capture nonlinear relationships in the data [43]. The linear SVM is a classification algorithm that identifies the optimal hyperplane to separate data points into distinct classes, making it effective for high-dimensional datasets [44].

### Feature Importance Estimation

Logistic regression is valued for its simplicity and interpretability [38]. Feature importance within this model is estimated by analyzing the coefficients. Larger absolute values of these coefficients suggest a stronger impact on the outcome, with positive coefficients increasing the log odds of the outcome (thus making it more likely), and negative coefficients decreasing the log odds (thus making it less likely). In our approach, we used an RFE algorithm, which selects varying numbers of features in each training fold. We first identified which features were consistently selected across all training folds. We then calculated the average coefficients for these features across all training folds and ranked them based on the absolute values of these averages. This method highlights the features that the model relies on to predict the likelihood of a patient’s improvement.

## Results

### Sample and Dataset Characteristics

The characteristics of the sample of TPS patient users of the MMP app who were included in the study and their records in the MMP app are shown in Table 1. Using an MCID of 2, 72 out of 160 (45%) of the patients showed improvements on the PROMIS PI questionnaire between the predictor and the outcome period. An overview of the PROMIS PI questionnaire response characteristics is shown in Table 2.

**Table 1.** Sample characteristics.

Category and variable		Value
Users		
Number of users		160
Age (years), mean (SD)		40.4 (16.6)
Age not provided		129
Gender (male)		14
Gender (female)		19
Gender not provided		127
Number of health conditions, mean (SD)		3.5 (3.4)
Health conditions not provided		90
Number of medications, mean (SD)		3.7 (3.3)
Medications not provided		58
MMP^a^ records		
Users with any MMP record in the predictor period		124
Total MMP records in the predictor period		4009
Users with a pain record in the predictor period		123
Number of pain records in the predictor period		2820
Users with a daily reflection in the predictor period		75
Number of daily reflections in the predictor period		1189
Pain record score in the predictor period, mean (SD)		5.1 (2.6)
Daily reflection score in the predictor period, mean (SD)		4.0 (2.7)
Total MMP records		18,545^b^
Total number of pain records		13,541^b^
Number of pain records per day per user, mean		0.3^b^
Total number of daily reflections		5004^b^
MMP days of activity, mean (SD)		411 (438)^b^

a MMP: Manage My Pain app.

b Values derived from total app use for each user.

**Table 2.** Characteristics of the PROMIS Pain Interference 8a v1.0 questionnaire responses.

Variable	Value
Total responses in predictor period	199
Total responses in outcome period	236
Mean days between first response and outcome response, mean (SD)	74.8 (39.4)^a^
Mean T-score in predictor period, mean (SD)	65.6 (6.7)
Mean T-score in outcome period, mean (SD)	63.1 (7.4)
Mean T-score change, mean (SD)	−2.5 (6.5)

a Range: 22‐183 days.

### Prediction Results

**Figure 3.**
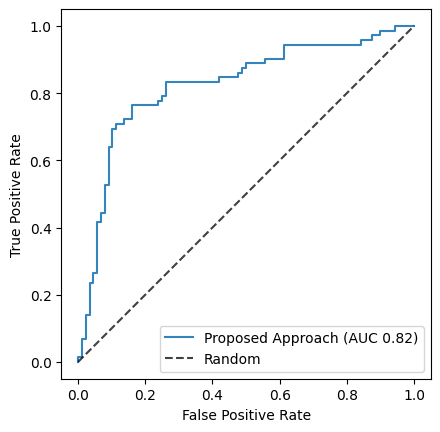
The receiver operating characteristic curve. The area under the receiver operating characteristic curve (AUC) is 0.82.

The model was evaluated on 160 subjects using leave-one-out verification. When evaluated without using RFE, including all features in the model, the accuracy was 74%. Including RFE improved performance by reducing overfitting. Using fewer features, the model had an overall accuracy of 79%, with an even performance across both improved and not improved classes. The accuracy for subjects that improved (sensitivity) was 76% and for subjects that did not improve (specificity) was 82%. The confusion matrix of the prediction results can be seen in Table 3. On average, the algorithm selected a mean of 88 (SD 13) out of the 245 features. The receiver operating characteristic curve is shown in Figure 3. The AUC was 0.82.

**Table 3.** Confusion matrix of the prediction results.

	Predicted improved class	Predicted not improved class	Total actual
Actual improved class	55	17	72
Actual not improved class	16	72	88
Total predicted	71	89	160

### Comparison

Three additional models were evaluated to compare against our chosen approach: AdaBoost, random forest, and linear SVM. To directly compare against the logistic regression model, the same prediction pipeline was used. First, the data were preprocessed, and then each model was trained with RFE to optimize the features. While all features were inputted into the pipeline, each model had different RFE optimizations across the training folds. Default hyperparameters were used for each of the models. Table 4 shows the results for each model. The logistic regression model consistently outperforms the other models across all metrics.

**Table 4.** Comparison of the logistic regression model to other models.

Model	Not-improved class accuracy	Improved class accuracy	Accuracy	AUC^a^
Logistic regression	0.82	0.76	0.79	0.82
Linear SVM^b^	0.74	0.67	0.71	0.75
Random forest	0.81	0.35	0.60	0.62
ADABoost	0.67	0.57	0.62	0.61

a AUC: area under the receiver operating characteristic curve.

b SVM: support vector machine.

### Feature Importance

In our study, a varying number of features were selected across 160 training folds, with an average of 88 out of 245 features chosen. Notably, 37 features consistently appeared across all folds. Table 5 displays the mean coefficients from the logistic regression model for these 37 features, indicating their importance in prediction.

**Table 5.** Mean coefficients from the logistic regression model for features that appeared across all training folds.

Rank	Feature	Mean coefficient
1	PCS^a^ question 1: “I worry all the time about whether the pain will end”	1.685
2	Body locations: Legs	1.440
3	PROMIS PI^b^ question 3: “How much did pain interfere with your ability to participate in social activities?”	1.437
4	PROMIS PI question 5: “How much did pain interfere with the things you usually do for fun?”	1.286
5	PROMIS PI question 1: “How much did pain interfere with your day-to-day activities?”	1.285
6	Meaningful activities: Exercised	1.253
7	Aggravating factors: Stress	−1.252
8	PCS question 2: “I feel I can’t go on”	−1.123
9	NRS^c^ pain question 3: “Please rate your pain by marking the box beside the number that tells how much pain you have right now”	−1.108
10	NRS pain score: Right now	−1.108
11	Effective factors: Massage	1.040
12	Ineffective factors: Talking to someone	1.027
13	Meaningful activities: “Connected with supportive people online or through text”	−1.000
14	Pain trend	−0.994
15	Number of conditions (Categories)	0.983
16	Locations: Neck	−0.979
17	PCS question 12: “There’s nothing I can do to reduce the intensity of the pain”	0.978
18	Locations: Head (Right)	−0.977
19	PHQ-9^d^ Question 7: “Trouble concentrating on things, such as reading the newspaper or watching television”	−0.892
20	Locations: Joints	0.891
21	Environment: Home	0.881
22	Medications: Tricyclic antidepressants	−0.880
23	GAD-7^e^ question 4: “Trouble relaxing”	−0.860
24	PCS question 5: “I feel I can’t stand it anymore”	−0.825
25	Symptoms: Insomnia	−0.825
26	Meaningful activities: Errands outside the home	−0.815
27	Pain characteristic: Custom entry	0.790
28	Height	0.789
29	Percent of completed descriptor elements	0.772
30	Mean time of day of pain record entry	0.746
31	Effective factors: Rest	0.736
32	Medications: Acetaminophen	−0.714
33	Conditions: Back pain	0.713
34	Environment: Work	−0.665
35	Effective factors: Ice	0.660
36	PROMIS PI score: Raw score	0.476
37	Symptoms: Dizziness	−0.41

a PCS: Pain Catastrophizing Scale.

b PROMIS PI: PROMIS Pain Interference 8a v1.0.

c NRS: Numeric Rating Scale.

d PHQ-9: Patient, Health Questionnaire-9.

e GAD-7: General Anxiety Disorder-7.

### Questionnaire Comparison

While this work was focused on optimizing a model for predicting improved scores on the PROMIS PI measure, we repeated the same algorithm for each of the questionnaires included in the dataset. As demonstrated in Table 6, responses on PROMIS PI had the best performance with an AUC of 0.81. Responses on GAD-7 and PHQ-9 showed some moderate ability for prediction, with AUCs of 0.64 and 0.74, respectively. Responses on GAD-7, PHQ-9, and PCS demonstrated lower rates of improvement and produced a more imbalanced dataset, possibly leading to decreased performance by the algorithm. Responses on the NRS for pain severity, on the other hand, indicated a similar percentage of improved MMP users as PROMIS PI. Despite the balanced dataset, the predictive model did not achieve any meaningful results, with an AUC of 0.51, which is nearly equivalent to random chance.

**Table 6.** Comparison of the logistic regression model performance on PROMIS Pain Interference questionnaire responses compared with the other questionnaires included in the dataset.

Questionnaire	MCID^a^	Number improved / Total Users, n/N (%)	Accuracy	Class 0	Class 1	Balanced accuracy	AUC^b^
PROMIS PI^c^ [22]	2 [37]	72/160 (45%)	0.79	0.82	0.76	0.79	0.82
GAD-7^d^ [34]	4 [45]	52/180 (29%)	0.70	0.70	0.71	0.70	0.64
PHQ-9^e^ [35]	5 [46]	34/169 (20%)	0.72	0.76	0.59	0.67	0.74
PCS^f^ [33]	38% [47]	47/173 (27%)	0.6	0.68	0.38	0.53	0.57
NRS^g^ [9]	1 [48]	89/173 (46%)	0.53	0.55	0.51	0.53	0.51

a MCID: minimal clinically important difference.

b AUC: area under the receiver operating characteristic curve.

c PROMIS PI: PROMIS Pain Interference 8a v1.0.

d GAD-7: General Anxiety Disorder-7.

e PHQ-9: Patient, Health Questionnaire-9.

f PCS: Pain Catastrophizing Scale.

g NRS: Numeric Rating Scale.

## Discussion

### Principal Findings

This study examined whether a machine learning model could predict clinical outcomes related to pain in a population of TPS clinic patients who used the MMP digital health solution to track symptoms and answer clinic questionnaires. Using MMP app data entered by patients for 30 days, a linear regression model predicted clinically significant improvement in pain interference measured by the PROMIS PI questionnaire with 79% accuracy. The model showed balanced accuracy between improved and not improved classes with a sensitivity of 0.76 and specificity of 0.82.

Analysis of the features used in the model showed that all MMP app data, not just questionnaire responses, were relevant in predicting patient improvement. This finding underscores the critical role of all types of data in the algorithm’s efficacy. Features like exercise showed a positive correlation with improved outcomes, while stress was negatively correlated, aligning with clinical expectations. However, many top-ranked features lacked such clinical clarity. This is not unexpected as the machine learning model integrates all features collectively to make predictions, preventing the isolation of individual feature impacts. Some features might be correlated with other variables that influence improvement, and they should not be interpreted independently in this type of prediction approach. Despite these complexities, our results affirm the value of leveraging extensive datasets, allowing the model to identify influential factors beyond traditional assumptions and providing a robust statistical foundation to determine the factors that predict improvement.

Predicting the development and progression of a chronic pain condition has important clinical implications. Currently, clinicians rely on known risk factors implicated in the development and progression of chronic pain conditions [49-55] to inform clinical decision-making regarding treatment and pain management. However, the individualized nature of chronic pain and the interacting contribution of physical, emotional, and social factors impose considerable challenges in accurately predicting patient outcomes [4,5]. Recent efforts have applied machine learning to large datasets to demonstrate that individualized pain risk scores can be determined from a set of biopsychosocial factors [5]. However, applying this approach in the clinical context is limited by the availability of relevant data for a specific patient population. This study bridges this gap by demonstrating that a pain-tracking app can be used in a real-world clinical setting to gather relevant data in a short period of time and effectively predict clinically significant outcomes related to pain. Information from the MMP app can be used by clinicians alongside traditional approaches to patient assessment to more effectively guide critical decision-making around medication management, such as tapering opioids, and allocation of finite clinic resources to patients with the greatest need. It is important to note that the approach presented here is not intended to identify specific predictors of improvement, but rather to help clinicians evaluate which patients are more or less likely to improve so they can prioritize health care resources accordingly.

### Limitations and Future Work

The findings presented here are limited to a small sample of a specific patient population who selected to use the MMP app, which may have contributed to a degree of selection bias in the dataset used for prediction modeling. Additionally, the dataset had many missing data points. Missing data frequently occur in real-world self-reported data sources, such as the one used here [56]. To replace missing values in our dataset, we relied on mean imputation. However, it is important to note that the missing data in the current dataset and the mean imputation approach may have contributed to unrecognized bias in the prediction model and affected the study outcome. Finally, only one clinical outcome is considered in the prediction model and further work is needed to identify how other clinically relevant outcomes can be incorporated into a more comprehensive prediction tool. Further refinement is also needed to increase the accuracy of the prediction model. The next steps in this ongoing work focus on integrating additional data from electronic patient records and facilitating greater engagement from patients with the MMP app. The current real-world use of the app was sufficient to achieve useful predictive insights, despite some caveats in their interpretation. Additional efforts to facilitate user engagement as well as data completion and integration will provide a richer dataset for the prediction algorithm to help improve its predictive capacity.

### Conclusion

This study builds on a growing body of work showing the capacity of pain apps like MMP to not only provide retrospective insights on symptom trends, but also serve as a clinical outcome prediction tool. Effectively predicting the progression of pain has the potential to improve clinical decision-making and personalized prevention and treatment of chronic pain. The findings of this study demonstrate that existing digital solutions like the MMP app offer a feasible approach to integrating patients’ self-tracking and clinical data in a machine-learning algorithm to develop accurate prediction models that can be used in a real-world clinical setting.

## Supplementary material

10.2196/67178 Multimedia Appendix 1 Clinic questionnaires.

## Abbreviations

AUC: area under the receiver operating characteristic curve
GAD-7: Generalized Anxiety Disorder-7 scale
MCID: minimal clinically important difference
MMP: Manage My Pain
NRS: Numeric Rating Scale
PCS: Pain Catastrophizing Scale
PHQ-9: Patient Health Questionnaire-9
PROMIS PI: PROMIS Pain Interference 8a v1.0 scale
RFE: recursive feature elimination
SVM: support vector machine
TGH: Toronto General Hospital
TPS: Transitional Pain Service
