# Supplementary material for: Predicting Clinical Outcomes at the Toronto General Hospital Transitional Pain Service via the Manage My Pain App: Machine Learning Approach
Source: JMIR Med Inform. 2025 Mar 28;13:e67178. doi: 10.2196/67178 (PMC11970568; doi:10.2196/67178)
Supplement: Multimedia Appendix 1 [file medinform-v13-e67178-s001.docx]

**Pain Numeric Rating Scale (NRS)**

1. Please rate your pain by marking the box beside the number that best describes your pain at its worst in the last 24 hours.

| 0 | 1 | 2 | 3 | 4 | 5 | 6 | 7 | 8 | 9 | 10 |
| --- | --- | --- | --- | --- | --- | --- | --- | --- | --- | --- |
| No Pain |  |  |  |  |  |  |  |  |  | Pain as bad as you can imagine |

1. Please rate your pain by marking the box beside the number that best describes your pain at its least in the last 24 hours.

| 0 | 1 | 2 | 3 | 4 | 5 | 6 | 7 | 8 | 9 | 10 |
| --- | --- | --- | --- | --- | --- | --- | --- | --- | --- | --- |
| No Pain |  |  |  |  |  |  |  |  |  | Pain as bad as you can imagine |

1. Please rate your pain by marking the box beside the number that best describes your pain on the average.

| 0 | 1 | 2 | 3 | 4 | 5 | 6 | 7 | 8 | 9 | 10 |
| --- | --- | --- | --- | --- | --- | --- | --- | --- | --- | --- |
| No Pain |  |  |  |  |  |  |  |  |  | Pain as bad as you can imagine |

1. Please rate your pain by marking the box beside the number that tells how much pain you have right now.

| 0 | 1 | 2 | 3 | 4 | 5 | 6 | 7 | 8 | 9 | 10 |
| --- | --- | --- | --- | --- | --- | --- | --- | --- | --- | --- |
| No Pain |  |  |  |  |  |  |  |  |  | Pain as bad as you can imagine |

Links to remaining clinic questionnaires for which user responses were included in the study dataset:

PROMIS Pain Interference 8a v1.0 (PROMIS PI)

<https://cde.nida.nih.gov/instrument/0a47dcdf-da3c-6b71-e050-bb89ad436524>

Patient Health Questionnaire-9 (PHQ-9)

<https://www.apa.org/depression-guideline/patient-health-questionnaire.pdf>

Pain Catastrophizing Scale (PCS)

<https://www.oregon.gov/oha/HPA/dsi-pmc/PainCareToolbox/Pain%20Catastrophizing%20Scale.pdf>

Generalized Anxiety Disorder-7 (GAD-7)

<https://adaa.org/sites/default/files/GAD-7_Anxiety-updated_0.pdf>
